# The Impact of Climatic Factors on Temporal Mosquito Distribution and Population Dynamics in an Area Targeted for Sterile Insect Technique Pilot Trials

**DOI:** 10.3390/ijerph21050558

**Published:** 2024-04-28

**Authors:** Theresa Taona Mazarire, Leanne Lobb, Solomon Wakshom Newete, Givemore Munhenga

**Affiliations:** 1 Centre for Emerging Zoonotic and Parasitic Diseases, National Institute for Communicable Diseases, National Health Laboratory Service, Johannesburg 2131, South Africa; theresama@nicd.ac.za (L.L.); givemorem@nicd.ac.za (G.M.); 2 Wits Research Institute for Malaria, School of Pathology, University of the Witwatersrand, Johannesburg 2050, South Africa; 3 Geoinformatics Division, Agricultural Research Council-Natural Resource and Engineering, Arcadia, Pretoria 0083, South Africa; newetes@arc.agric.za; 4 School of Animal, Plant and Environmental Sciences, University of the Witwatersrand, Bramfontein, Johannesburg 2050, South Africa

**Keywords:** malaria, *Anopheles arabiensis*, ARIMA, climate, temporal distribution, Mamfene area, sterile insect technique, time series, temperature

## Abstract

It is widely accepted that climate affects the mosquito life history traits; however, its precise role in determining mosquito distribution and population dynamics is not fully understood. This study aimed to investigate the influence of various climatic factors on the temporal distribution of *Anopheles arabiensis* populations in Mamfene, South Africa between 2014 and 2019. Time series analysis, wavelet analysis, cross-correlation analysis, and regression model combined with the autoregressive integrated moving average (ARIMA) model were utilized to assess the relationship between climatic factors and *An. arabiensis* population density. In total 3826 adult *An. arabiensis* collected was used for the analysis. ARIMA (0, 1, 2) (0, 0, 1)_12_ models closely described the trends observed in *An. arabiensis* population density and distribution. The wavelet coherence and time-lagged correlation analysis showed positive correlations between *An. arabiensis* population density and temperature (r = 0.537 ), humidity (r = 0.495) and rainfall (r = 0.298) whilst wind showed negative correlations (r = −0.466). The regression model showed that temperature (*p* = 0.00119), rainfall (*p* = 0.0436), and humidity (*p* = 0.0441) as significant predictors for forecasting *An. arabiensis* abundance. The extended ARIMA model (AIC = 102.08) was a better fit for predicting *An. arabiensis* abundance compared to the basic model. *Anopheles arabiensis* still remains the predominant malaria vector in the study area and climate variables were found to have varying effects on the distribution and abundance of *An. arabiensis*. This necessitates other complementary vector control strategies such as the Sterile Insect Technique (SIT) which involves releasing sterile males into the environment to reduce mosquito populations. This requires timely mosquito and climate information to precisely target releases and enhance the effectiveness of the program, consequently reducing the malaria risk.

## 1. Introduction

For decades, malaria has posed a health burden [1], with approximately 249 million cases in 85 malaria-endemic countries in 2022, a 5 million increase from the previous year [2]. About 94% of these global cases occurred in the WHO African Region, with South Africa contributing around 2000 malaria cases in 2022 [2]. Malaria transmission in South Africa is primarily driven by *Anopheles arabiensis*, the main malaria vector [3,4], along with other potential secondary vectors, *An. vaneedeni*, *An. parensis* and *An. merus* [5], after the near eradication of *An. funestus* (sensu stricto) using intensive Indoor Residual Spraying (IRS) [6].

Despite the progress in malaria vector control, indigenous transmission still persists in the endemic provinces [2,7], challenging the 2030 malaria elimination agenda [3,8,9]. Current vector control strategies such as the IRS supplemented by winter larviciding are the main vector control strategies in South Africa [3,5]. However, these are facing challenges such as insecticide resistance, environmental effects, and cost-effectiveness of these approaches under a low transmission setting [5]. In addition the exophagic/exophilic behavior of the primary vector, *An. arabiensis* is presenting a further complication [9]. IRS is primarily designed to target indoor biting and resting mosquitoes but is not fully effective against vectors such as *An. arabiensis* which has cosmopolitan feeding and resting behavior. Consequently, complementary strategies like the Sterile Insect Technique (SIT) are being explored. The SIT involves mass production, sex separation, sterilization, and release of sterile males with the hope that they will mate with wild females and produce unviable offspring [10].

The SIT is a targeted precision-based control strategy that heavily relies on a comprehensive understanding of the distribution of the targeted species. The effectiveness of this technique is highly dependent on understanding the intricate factors that determine the temporal population distribution and dynamics to guide decisions on the optimal timing and location for releasing the sterile mosquitoes [11,12]. Although South Africa has made significant strides in testing the feasibility of SIT and is now in the pilot field trial phase [11,13,14,15,16,17], it does not have enough baseline data on the relationship between mosquito occurrence and climate to guide sterile male releases.

Climate is one of the factors that play a fundamental role in determining mosquito populations’ survival, reproduction, growth, abundance, dispersal, and distribution [18]. Various studies have been conducted to understand the influence of different climatic factors on mosquito population dynamics particularly the impact of temperature, rainfall, wind, and humidity on mosquito distribution [19,20,21,22,23,24]. Climate can affect mosquito populations directly or through influencing land and atmospheric conditions of mosquito ecosystems. The climate varies over time for different geographical areas and can also vary in the same geographical area, creating unique heterogeneous macro-spatial landscapes and microclimate niches for mosquitoes. Mosquito species distribution and their population temporal dynamics are usually organized based on the favorable micro-climates for each species [25]. Therefore, rainfall, temperature, wind, and humidity play a significant role in influencing the distribution of mosquitoes and controlling their population in time and space.

Rainfall is responsible for creating and or sometimes destroying aquatic habitats that act as oviposition sites for females and ecological niches for mosquitoes’ aquatic stages. Mosquito abundance is closely linked to the quantity of rainfall received, with variations and amounts notably affecting their prevalence [21,26]. Rainfall, if seasonal, drives the occurrence of breeding sites which in turn affects the seasonality and temporality of mosquito distribution. On the other hand, the occurrence of harsh, heavy rains can hinder the oviposition of eggs and wash away existing larvae, as well as transform mosquito aquatic habitats into rivers that are not suitable for mosquito oviposition [27]. Furthermore, heavy rain sometimes causes the mortality of adult mosquitoes impacting population density.

In addition to rainfall, temperature is a critical determinant of mosquito population dynamics since mosquitoes rely on the moisture and warmth in the atmosphere. As poikilotherms, mosquitoes’ development in each of their life stages is dependent on temperature [28,29]. Temperature influences physiological functions such as longevity, biting rates, larval and adult development, susceptibility to insecticides, reproduction, gonotrophic cycles, fecundity, survival, and general behavioral characteristics [21,22,29]. An increase in temperature accelerates mosquito development as well as increased biting and feeding frequency. For example, [30] demonstrated that warmer temperatures between 26 °C and 29 °C tend to increase blood feeding frequency and subsequent blood digestion leading to higher fecundity. However, this does not directly result in an increased transmission potential as there is also a direct relationship between temperature and parasite development inside the mosquito body. The rate at which the malaria parasite (*Plasmodium*) matures inside the mosquito notably declines as temperature rises [31], reducing transmission potential. On the contrary warmer temperatures encourage faster maturation of the mosquito larvae [32], increasing the developmental time.

Besides temperature and rainfall, humidity has been shown to play an important role in determining the position that mosquito species take within an ecosystem [33]. Several studies proved that humidity influences blood-feeding intervals, mating, longevity, dispersal, survival, and egg oviposition of mosquitoes [33,34,35,36,37]. However, the level of humidity required by each mosquito varies with some mosquito species favoring high humidity (relative humidity–RH above 70%) while some studies have shown that species such as *An. arabiensis* have adapted to drier air (below 65% RH) [38]. However, studies on mosquito desiccation have shown that mosquitoes find it difficult to survive when exposed to extremely low relative humidity for longer periods [33,39].

Another climatic variable that is known to play a role in shaping the micro-spatial distribution of mosquitoes is wind [20,34,40]. Wind, particularly the direction of the wind, causes the advection of adult mosquitoes by carrying the scents that attract them to their blood meals or plants’ nectar for energy sources [41,42]. The direction in which mosquitoes fly toward odor sources is determined by both the temporal and spatial distribution of odorants downwind [43], hence influencing mosquito distribution in space. Additionally, the wind indirectly affects the mosquito population by affecting larval survivorship. Wind causes water waves around breeding sites, if wind speeds are high they reduce larval survival [41].

The relationship between specific climatic envelopes and mosquito population dynamics cited above highlights the critical role of climate, in determining mosquito population distribution. In South Africa, climate has been documented to be the main contributor to the distribution of malaria vectors [20,21,23,24,26,31]. However, to the best of our knowledge, there is no information on the distribution of *An. arabiensis* at a microscale particularly in Mamfene, northern KwaZulu-Natal an area targeted for SIT pilot releases. While previous studies have detailed the general population dynamics of *An. arabiensis* in Mamfene [11], there is a limited understanding of the climatic drivers of population dynamics that can guide when and how sterile male releases should be conducted. This study used the ARIMA model to determine how climatic factors influence the temporal distributions of *An. arabiensis* in Mamfene. The information generated facilitate the planning of the implementation of SIT pilot trials.

## 2. Materials and Methods

### 2.1. Study Area

**Figure 1:**
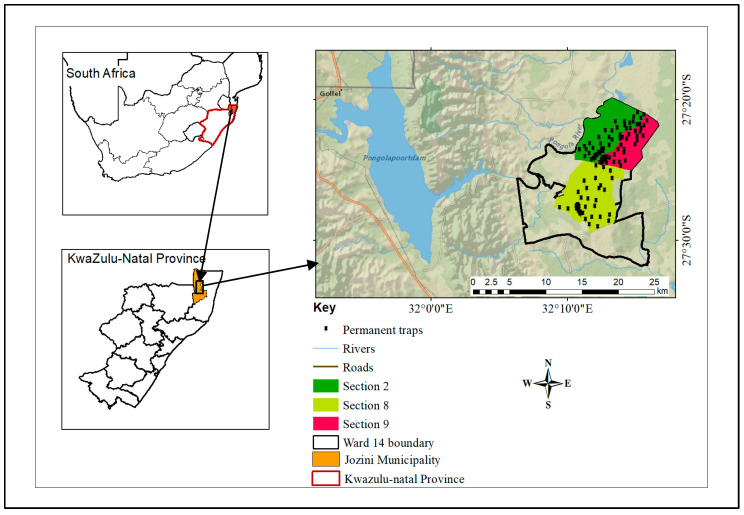
Map showing the location of the study area Mamfene, UMkhanyakude District Municipality in northern KwaZulu-Natal, South Africa. The arrow points to Mamfene and the three mosquito sampling sites, i.e., Sections 2, 8, and 9, are represented by blue, green, and yellow shading, respectively.

The study was conducted in the Mamfene, Jozini Municipality, northern KwaZulu-Natal, South Africa (“27°20′17.95″ S; 32°12′53″ E) (Figure 1). The area is located to the east of Pongolapoort Dam and adjacent to the western border of uMhlabuyalingana Local Municipality. The study focused on Sections 2, 8, and 9 that are in Ward 14, (Figure 1) because of comprehensive entomological and climatic data which have been consistently gathered over 9 years as part of an SIT feasibility assessment. These sections fall within the KZN malaria control program entomological surveillance sentinel sites [17]. All three sections cover an area of approximately 13,000 hectares.

The area encompasses a wetland spanning Sections 2, 8, and 9, covering an area of about 600 hectares which provides perennial mosquito breeding sites. The local community predominantly cultivates maize, sugarcane, and pumpkins. Additionally, they rear domestic animals, including cattle, goats, pigs, dogs, and chickens providing potential blood-feeding sources for mosquitoes. The land cover is diverse, including sparsely dense forestry with mostly indigenous trees that provide shade and extrafloral nectaries, as well as fruits that provide an energy source for mosquitoes. The climate in the area is subtropical, with an average annual temperature and rainfall of 21 °C and 621 mm, respectively. Mamfene receives its rainfall from mid-summer to the onset of autumn, with most of the rainfall being received in March which coincides with the peak malaria transmission season. The coldest months are June and July, with February being the hottest month, with an average of 33.56 °C [44].

### 2.2. Data Collection

Both mosquito and climate data used in this study span over 6 years from February 2014 to December 2019.

#### 2.2.1. Mosquito Data

Regular entomological surveillance has been going on at the three SIT sentinel sites (Sections 2, 8, and 9) (Figure 1) since January 2014 and is still ongoing. Live adult mosquito specimens are collected approximately twice a week (i.e., eight times per month) from clay pots and modified plastic buckets permanently stationed close to human settlements and potential breeding site points (with prior owners’ consent) [9,11]. Collections from these traps were carried out from 05:30 hrs to 09:00 hrs. Additionally, mosquitoes are occasionally collected from disused tires, drums, carbon dioxide-baited net traps, and direct aspiration from cattle kraals.

Adult mosquitoes used for this study were first identified morphologically in the field laboratory using a dichotomous key [45]. Positively identified specimens were sent to the Vector Control Laboratory at the National Institute for Communicable Diseases (NICD) in Johannesburg for further identification at the species level using polymerase chain reaction (PCR) protocol according to the methods of [46,47]. Subsequently, an analysis of other entomological indicators was conducted. The data collected included the global positioning system (GPS) location data of each mosquito trap where mosquitoes were collected, along with its corresponding trap number, and date of collection. These data stored in the SIT database were retrieved at the beginning of this study.

**Figure 2:**
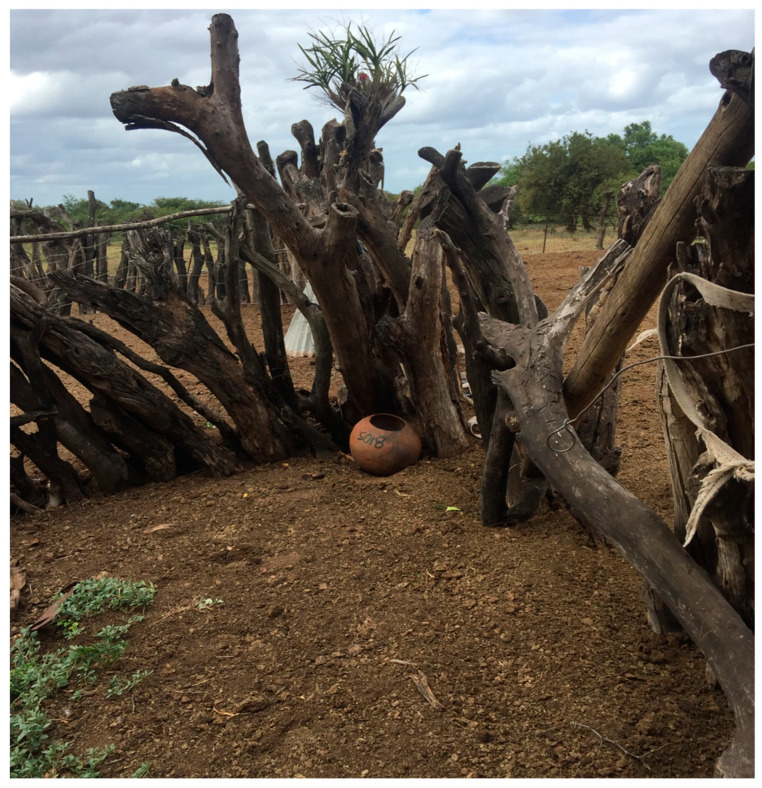
Outdoor resting trap (clay pot) used to collect *Anopheles* mosquitoes in the Mamfene area, KwaZulu-Natal Province in South Africa.

Table 1 summarizes the species of all mosquitoes collected in the study area between 2014 and 2019. The present study focuses on the malaria vector species *An. arabiensis* and is limited to data collected from clay pots (Figure 2). This was because *An. arabiensis* constitutes the primary malaria vector and data from clay pots was consistent throughout the study period. To facilitate analysis, data for *An. arabiensis* was categorized into seasons to explore if there was any form of seasonality. A new variable, “season”, was computed and incorporated into the analysis. This variable was generated with specific date ranges for each season, as outlined below:

- Summer: 1 December to 29 February
- Autumn: 1 March to 31 May
- Winter: 1 June to 31 August
- Spring: 1 September to 30 November

#### 2.2.2. Climate Data

Meteorological data consisting of various climatic variables, including average daily minimum and maximum temperature which was aggregated into mean temperature (°C), monthly daily rainfall (in mm), monthly average of the wind speeds (m/s), and monthly average humidity (%), were used in the analysis. The data were obtained from the South African Weather Service (SAWS) meteorological station located at Makhatini Research Centre which is in Section 2 within the study area. Data were recorded monthly in a spreadsheet.

### 2.3. Data Analysis

#### 2.3.1. Descriptive Statistics

The mosquito dataset retrieved included mosquito species, location of collection, date of collection, and collection trap type. The number of *An. arabiensis* collected in each section over the six years was summarized as the relative frequencies and stratified by year and season. The mosquito dataset included mosquito density data which were determined by the number of *An. arabiensis* collected from the clay pots and sampling effort, i.e., the number of times a pot was visited for mosquito collection in a given month for each section. The Box–Jenkins approach was used to perform a time series analysis. Monthly temporal patterns and seasonal variation of *An. arabiensis* density was analyzed using decomposing time series analysis. An additive model was developed from the observed constant seasonal variation, which did not increase over time. The additive decomposition was used because of its suitability when seasonal fluctuations and trend-cycle variations remained consistent regardless of the time series level [48]. The time series additive model used is given in Equation (2) below.

Equation (1): Time series additive model

*Y[t]* = *T[t]* + *S[t]* + *e[t]* (1)

where *Y[t]* is the observed series at time *t*, *T[t]* is the trend-cycle component at time *t* (which includes cyclical and longer trend patterns, the “trend-cycle” component), *S[t]* is the seasonal component at time *t*, *e[t]* is the remainder/irregulars component at time *t* (residuals after removing seasonal and trend components) [49].

The trend-cycle component was analyzed using linear regression. The model used the moving averages and ‘seasonal and trend decomposition according to the loess’ method to smooth and decompose the time series. Autoregressive Integrated Moving Average (ARIMA) modeling approach was then employed to analyze *An. arabiensis* density data and to assess autocorrelations and forecast future time series trends.

Equation (2): The fFormula used to assess autocorrelations and forecast future time series trends for the *Anopheles arabiensis* density data

(2) $$ARIMA=\left(p,\;d,\;q\right)(P,D,\;Q)m$$

ARIMA has an autoregressive component which refers to the number of lagged observations in the model, with “*p*” representing the number of lagged values in the non-seasonal part while “*P*”, represent the order in the seasonal lags. The model also has an intergrated component which refers to the degree of differencing needed to make the time series stationary where “*d*” represents the order of non-seasonal differences, and the “*D*” is the seasonal differencing. The last component of the ARIMA is the moving average which is the number of lagged errors in the forecast model whereby “*q*” represents the order of lagged errors in the non-seasonal part and “*Q*” is the order of lagged errors in the seasonal part of the model. The term “*m*” refers to the number of time-series observations in a seasonal cycle [50].

The Akaike Information Criterion (AIC) was used to determine the best-fit model to proceed to the dynamic regression analysis process.

#### 2.3.2. Wavelet Coherence Analysis

To investigate the interconnectedness between the climatic variables and *An. arabiensis* mosquito density, wavelet coherence analysis was utilized. This approach has been recognized as the most effective technique for analyzing nonstationary data [24,26,51] where time intervals and frequency bands can be detected where two-time series show correlation. It extracts pertinent information from frequency fluctuations while identifying significant local temporal patterns such as sudden peaks and gaps [52].

The method uses various scales to analyze different frequencies. Consequently, wavelet transformation offers superior frequency resolution and less precise time resolution at lower frequencies. Building upon the Fourier analysis, the univariate wavelet power spectrum can be extended to assess statistical associations between two-time series *x(t)* and *y(t)* through wavelet coherence computation, which is formulated as follows:

Equation (3): Wavelet coherence

(3) $$Rx,y\;\left(f,\;\tau \right),=\frac{\left|\langle Wx,y\left(f,\;\tau \right)\rangle \right|}{\left|\;\langle Wx\left(f,\;\tau \right)\rangle \right|1/2|\langle Wy(f,\;\tau )\rangle 1/2\;}$$

The notation 〈〉 indicates smoothing in both time and frequency, where *Wx(f*, *τ*) represents the wavelet transform of series *x(t)*, *Wy(f*, *τ)* represents the wavelet power transform of series *y(t)*, and *Wx*,*y(f*, *τ)* represents the cross-wavelet power spectrum. The wavelet coherence offers localized insights into the degree of linear correlation between two nonstationary signals *x(t)* and *y(t)* at a specific period or frequency. *Rx*,*y(f*, *τ)* equals 1 when a perfect linear relationship exists between the two signals at a particular time and frequency [53].

In this study, wavelet analysis was used to understand coherence and time-phase lag between the mosquito density and climatic variable series as a function of both time and frequency. The results were then complemented with time-lagged correlations and a dynamic regression model. The visual analysis of the wavelet cross coherence allows checking in which time intervals, the associations are significant. Monte Carlo methods are employed to estimate the statistical significance level of the wavelet coherence [54]. The analysis was conducted in R using the *biwavelet* package [55].

#### 2.3.3. Model Fitting and Forecasting

The climatic variables were lagged at 0 to 3 months before and after to determine the maximum significant positive correlations. Pearson’s correlation coefficient was calculated to determine associations between temporally lagged monthly climatic variables and *An. arabiensis* density. The lags with the highest positive correlations were incorporated into the dynamic regression model to determine the relationship between climatic factors and *An. arabiensis* density and to explain historical variations. The *p*-values were then calculated to determine the significant predictors to forecast *An. arabiensis* population density. The significant lags were incorporated into the dynamic ARIMA model as external regressors. The errors from the regression model were allowed to contain autocorrelation from the best-fit ARIMA model. The R function *Arima()* was used to fit a regression model with ARIMA errors. The difference was applied to all variables in the regression model before the model was estimated. To forecast the *An. arabiensis* abundance, the ARIMA model utilized the significant lagged predictors. The model adequacy and ARIMA predictive model were assessed by using AIC. Statistical analysis was carried out using Stata/SE 14.2 [56] and R 3.6.3 [57]. *p* < 0.05 and 0.1 were considered as statistically significant.

## 3. Results

### 3.1. Descriptive Statistics

#### *Anopheles arabiensis* Population Dynamics

A total of 20,714 mosquito specimens were collected between 2014 and 2022, of which 3826 were *An. arabiensis* collected using clay pots (Table 1). The years 2019 (41.6%) and 2016 (23.6%) recorded the highest numbers of *An. arabiensis* collections, with densities of 12.9 and 13.7, respectively. The lowest collections were observed in 2014 (6.1%), 2018 (6.5%), 2017 (9.2%), and 2015 (12.9%), which had densities of 14, 5.6, 13.7, and 8 respectively. Approximately 43.6% (n = 1667), 38.8% (n = 1486), and 17.6% (n = 673) were collected in Sections 9, 2, and 8 respectively translating to mosquito densities of 22.8, 28.7, and 16.4 mosquitoes per clay pot. Significant variations in mosquito density were observed between seasons, with the highest percentage of *An. arabiensis* collected in summer (33.7%, n = 1291) and autumn (27.3%, n = 1043) with a total density of 17.2 and 25.8, respectively, for all the sections. The lowest collections were observed in spring (15.6%, n = 598) and winter (23.4%, n = 894) with total densities of 13.9 and 10.9, respectively, for all the sections. Overall, the highest proportion of *An.arabiensis* was collected from Section 9 with 44% (n = 1667), followed by Section 2 with 39% (n = 1486) and Section 8 with the lowest collection of 18% (n = 673) (Table 2).

**Figure 3:**
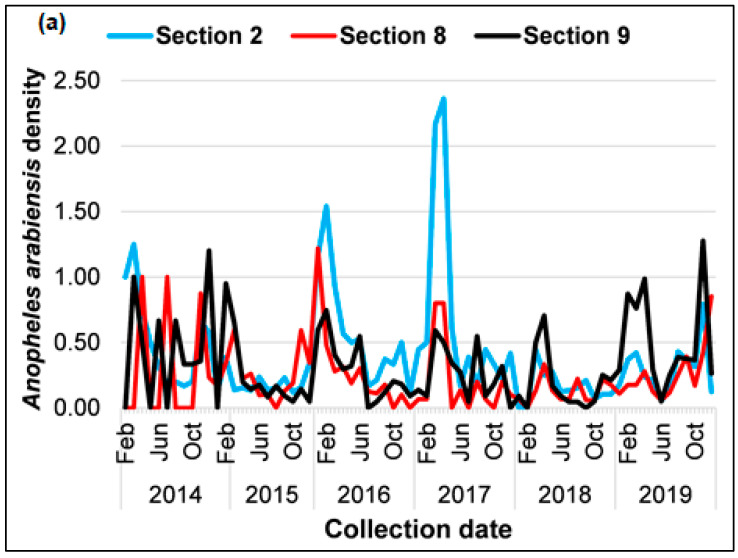

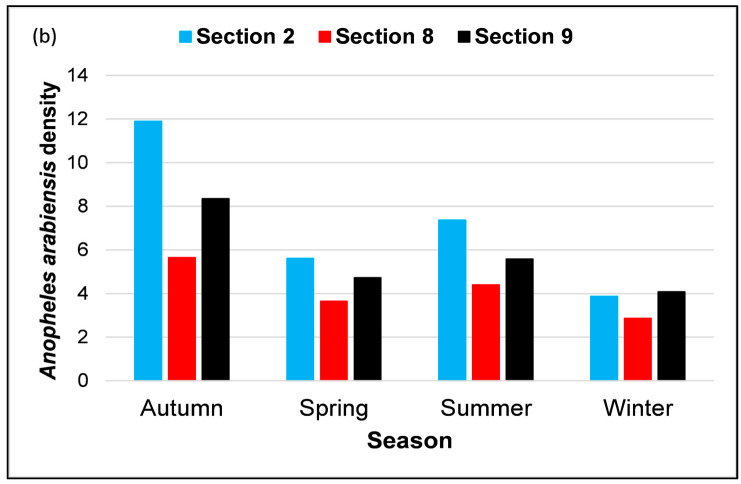
The population of *Anopheles arabiensis* mosquitoes collected using clay pots from Mamfene, KwaZulu-Natal between January 2014 and December 2019 stratified by the sections across different years (**a**) and seasons (**b**).

The results of the temporal annual baseline population dynamics of *An. arabiensis* showed their density was consistent throughout the years 2014 to 2016 compared to the years 2017 to 2019 where spikes and fluctuations in all three sections were observed (Figure 3a). The first peak occurred from January to March 2016, while the second and highest peak was observed from January to April 2017 in Section 2 and Section 8 (Table 2). There was also a decline in mosquito density from April to September of 2015 in Sections 8 and 9. Figure 3b shows the aggregated seasonal trends throughout the study period. The highest collections were recorded in autumn for Sections 2, 9, and 8, respectively. Overall, Section 2 had the highest mosquito density throughout the study period and in all seasons (summer y = 7.3, winter y = 3.9, autumn y = 11.9, and spring y = 5.6), while Section 8 had the lowest density throughout all the seasons (summer y = 4.4, winter y = 2.9, autumn y = 5.6 and spring y = 3.6) (Table 2).

### 3.2. Time Series of Anopheles arabiensis Density and Climatic Variables

**Figure 4:**
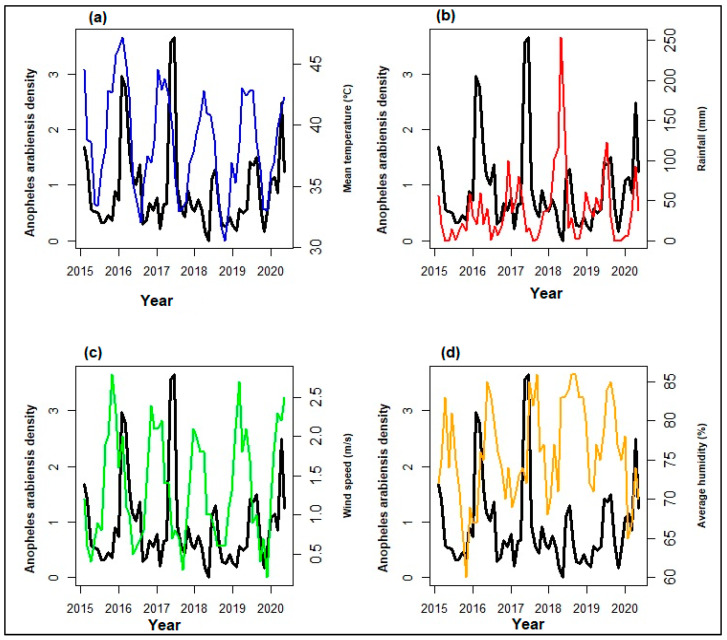
Time series plots showing the *Anopheles arabiensis* density plotted on the left *Y*-axis with its trend indicated using the black line and each of the climatic factors (**a**) mean temperature-blue (**b**) rainfall-red (**c**) wind speed-green (**d**) average humidity-yellow on the right *Y*-axis with their trend indicated using the colored lines for Mamfene, KwaZulu-Natal from 2014 to 2019.

The analysis showed no significant difference in *An. arabiensis* density between sections (*p* value = 0.119). Based on this analysis, the density data in the three sections were then merged for further analysis. There were high peaks in mean temperatures observed throughout the years with the highest temperature values observed in summer (maximum value = 35.6 °C) and lower values (minimum value = 9.3 °C) in winter. The rainfall season was observed in the spring and autumn months while the low rainfall was observed in the winter months. The rainfall fluctuated extensively showing a peak in February 2018 (253 mm) with a noticeable decline in June in the years 2014, 2015, 2017, and 2019. Wind speed was consistent throughout the years, although it showed cyclical patterns with high observation from October to January each year while the low values of wind speed were observed from April to August. The same was true for humidity which was consistent throughout the years ranging from 60% to 86%. In general, all series presented several peaks and fluctuations (Figure 4). Both *An. arabiensis* density and climatic time series from 2014 to 2019 exhibited seasonal peaks from January to March of the year and declines in the middle of the year during winter for all the years. The seasonal peaks of both mosquito density and climate in the series are separated by more than a few months indicating a cyclical pattern. However, the trends were not uniform throughout the years, except for the mean temperature.

### 3.3. ARIMA Modelling of the Anopheles arabiensis Density

The *An. arabiensis* density data were transformed into a time series and broken down into trend-cycle (*Tt*), seasonal (*st*), and error residual (*Et*). The density showed a declining trend, as demonstrated by the linear regression test, (*y* = 97.89588 − 0.04808 × beta, *p* < 0.05, R squared = 0.01302). There was a structural break in 2014 (*S* = 0.24941, *p*-value = 0.9635). However, this was not significant, and there was no strong evidence of structural changes in the time series data.

**Figure 5:**
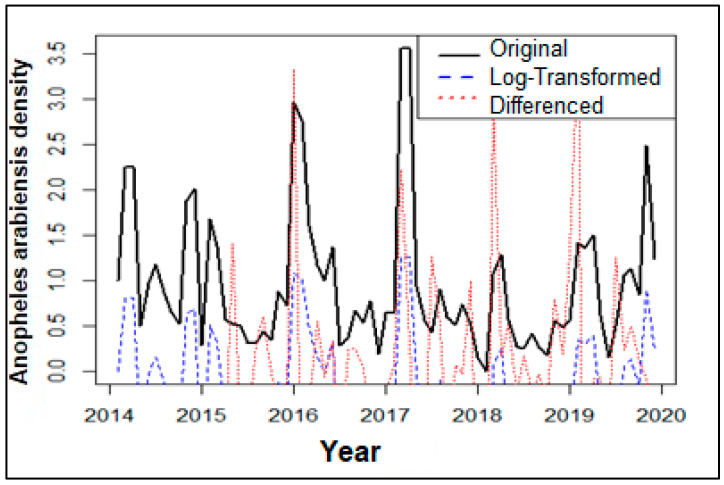
*Anopheles arabiensis* density of specimens collected between 2014 and 2019. Trend presented in black represents data before transformation, blue represents density after log transformation, and red present density after differencing.

The original time series showed increasing variability in *An. arabiensis* density along with a slightly increasing trend, suggesting that the time series needs both the non-seasonal and seasonal differencing to be stationary. To stabilize the variance and remove the linear trend, a log transformation followed by differencing was applied to the time series (Figure 5).

**Figure 6:**
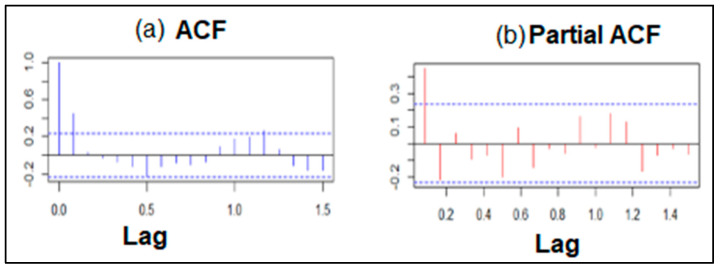
Autocorrelation-blue spikes (**a**) and partial autocorrelation-red spikes (**b**) of *Anopheles arabiensis* density time series data. The dashed lines show the significant threshold which is at a 95% confidence interval.

The temporal dependence structure of the time series was determined by analyzing the autocorrelation (ACF) and partial autocorrelation (PACF) plots. Based on the ACF and PACF plots (Figure 6a and Figure 6b, respectively), an ARIMA model of order (0,1,2) (0,0,1)_12_ was suggested.

Other models were also explored using data from 2014 to 2018. Among these models, ARIMA (0,1,2) (0,0,1)_12_ had the lowest AIC (125.28) and MAPE (74.80). This was confirmed to be the best model to fit the time series of *An. arabiensis* population.

### 3.4. Relationship between Anopheles arabiensis Density and Climatic Variables

**Figure 7:**
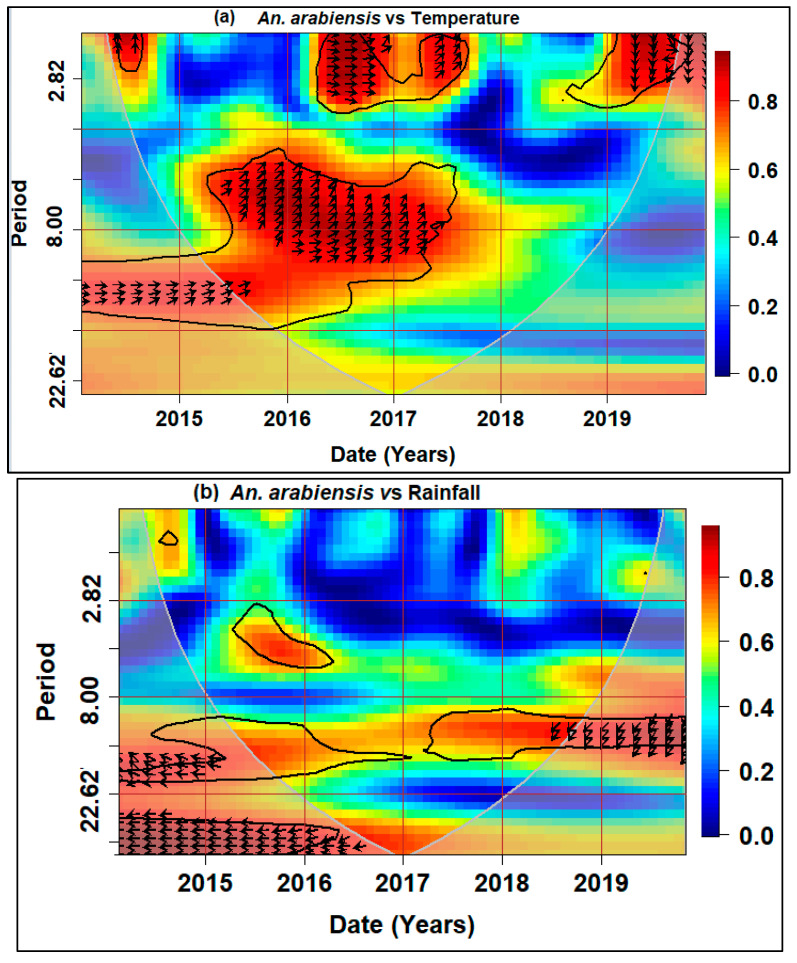

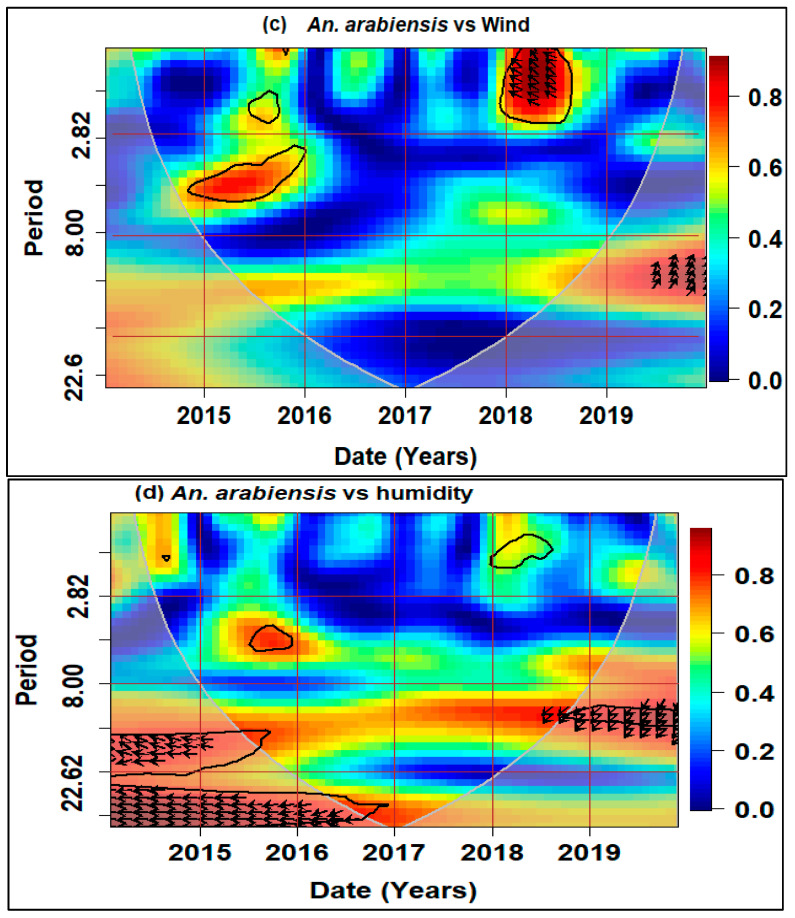
Wavelet cross-coherence of *Anopheles arabiensis* density and (**a**) mean temperature, (**b**) rainfall, (**c**) wind speed, (**d**) average humidity of Mamfene area in KwaZulu-Natal, South Africa, from 2014 to 2019. The *x*-axis shows time and the *y*-axis shows frequency of period value. The key bar shows frequency values (the lower values with dark colors show lower periods of co-movement and the higher values shown by warm colors represent higher periods of co-movement between variables.

The relationship between the *An. arabiensis* density and climatic variables based on the wavelet spectrum analysis are shown in Figure 7. Warmer colors (red) shows regions with strong interrelation whereas the colder colors (blue) indicate less dependence between the series. The lead/lag phase relations between the series are shown as an arrow in the wavelet coherence plots. When there is no phase difference, the two time series move together on a specific scale. When the time series are in phase, arrows point to the right and when they are out of phase arrows point to the left. When two series are in phase, they are moving in the same direction; when they are out of phase, they are moving in the other direction. The first variable is leading when arrows are pointing to the right-down or left-up, while arrows pointing to the right-up or left-down show that the second variable is leading [52].

Significant coherence was observed for the 4–10 period bands for mean temperature with an in-phase relationship, i.e., positive correlation. The in-phase (right arrows) indicates that the increase in mean temperature leads to *An. arabiensis* occurrence meaning that *An. arabiensis* occurrence is influenced by temperature. The overall coherence in this band was higher than 0.8.

For the rainfall, intensity was observed in the earlier years (2014–2016) and 2019. The coherence region was between 10 and 22 period bands. The antiphase relationship (left arrows) indicated a negative correlation with *An. arabiensis* density.

Wind showed lesser dependence with *An. arabiensis* with a small antiphase relationship indicating a negative correlation between these two time series.Humidity showed a very low correlation for the period 4–6 band and an with an anti-phase relationship from 2014 to 2017 while going onwards it showed that humidity is leading and the relationship with *An. arabiensis* density was weak.

These relationships were investigated further to determine the specific months when the climatic variables correlated with *An. arabiensis* density. The correlation between *An. arabiensis* density and climatic variables varied across the different lags. The mean temperature was significantly correlated with *An. arabiensis* density at −1 month lag (r = 0.433), lag 0 (r = 0.537), 1-month lag (r = 0.477) and 2-month lag (r = 0.473). The positive relationship between mean temperature and *An. arabiensis* density shows that an increase in more days with high temperatures leads to an increase in *An. arabiensis* density. Rainfall exhibited weak correlations at −1 lag month (r = 0.298), 1-month lag (r = 0.247), and 2-month lag (r = 0.235) whilst lag 0 (r = −0.155) showed a negative correlation. Negative correlations occurred with the wind at lag 0 (r = −0.466). There were positive correlations with humidity at lag 0 (r = 0.495), 1-month lag (r = 0.478), and 2-month lag (r = 0.456) (Table 3). Overall, the maximum positive/negative correlations between *An. arabiensis* density and the climatic variables occurred at lag 0 for mean temperature, lag −1 for rainfall, lag 0 for wind and lag 0 for humidity.

**Figure 8:**
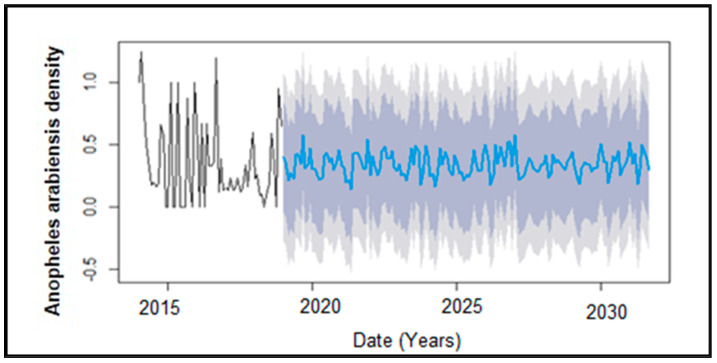
The original and forecasted monthly *Anopheles arabiensis* density time series in Mamfene, northern KwaZulu-Natal, South Africa, for the period 2014 to 2019. The black line represents the original time series mosquito density (2014–2019) and the blue line is the forecasted mosquito density between 2020 and 2030. The dark gray shaded color indicated the Lo 95% confidence interval and the light gray Hi 95% confidence interval of the predicted mosquito density values.

The lags with the highest recorded correlations were considered for regression analysis (lag 0 mean temperature, lag −1 rainfall, lag 0 wind, lag 0 humidity). The regression results for all the variables showed lag-1 rainfall (*p* = 0.0029), and lag-0 humidity (*p* = 0.0103) to be significant, whilst lag0 mean temperature (*p* = 0.254) and lag-0 wind (*p* = 0.2028) were insignificant to *An. arabiensis* density at 0.05 and 0.1 significant levels. When individual tests were run for each climatic variable against *An. arabiensis* density, the results showed lag 0 temperature (*p* = 0.00119), lag-1 rainfall (*p* = 0.0436) lag 0 humidity (*p* = 0.0441) to be significant predictors whilst lag0 wind (*p* = 0.770070) remanined an insignificant predictor. The significant lagged values of the climatic variables were used as predictors in the ARIMA model to help forecast future mosquito populations. This dynamic regression ARIMA model demonstrated a lower AIC (102.08), compared to the basic ARIMA baseline model (AIC = 125.28) indicating that the model provides better predictions than a basic ARIMA approach. The dynamic regression ARIMA model had better long-term predictive power compared to the ARIMA model with no external regressors. Figure 8 shows that the forecasted *An. arabiensis* density abundance will have seasonal peaks considering that all the climatic factors are favourable.

## 4. Discussion

It is crucial to exactly understand the role of climate in driving the microscale temporal distribution of mosquito species, particularly vector control approaches such as SIT which rely on precise knowledge of species location in time and space to enhance targeted resource allocation [10,58]. Despite knowledge about *An. arabiensis* population dynamics and its role in malaria transmissions in the Mamfene region, there remains a limited understanding of what influences mosquito abundance and the possible role that climate plays in driving the temporal population dynamics. In this work, a dynamic regression model which includes an ARIMA model incorporating external regressors was used to explain the historical variation and temporal dynamics of mosquitoes based on the climatic variables. Overall, the model developed indicated that the different climatic variables like temperature, rainfall, humidity, and wind speed have complex and sometimes opposing effects on mosquito populations over time, which were not always linear. Using the surveillance data collected between 2014 and 2019, this study illustrated the temporal patterns of *An. arabiensis* and investigated the effect of climatic factors on *An. arabiensis* population dynamics. *Anopheles arabiensis* was the dominant member of the *An. gambiae* complex over the 6-year sampling period, hence it was the focus of this study.

### 4.1. Anopheles arabiensis Population Temporal Dynamics

The abundance of *An. arabiensis* was consistent throughout the years, aligning with earlier studies conducted in the Mamfene, KwaZulu-Natal [9,11]. The highest total collections of *An. arabiensis* were recorded in 2019, attributed to high sampling effort, while 2014 had the least collections due to limited sampling effort as mosquito surveillance started in February of that year [11].

The *An. arabiensis* density showed seasonal variations, peaking in autumn and spring and declining in winter, consistent with previous studies [11,20], where seasonal variations were also observed. The high mosquito numbers in summer might be a result of warmer temperatures which encourage faster gonotrophic activities as well as a faster developmental rate of aquatic stages [59]. Warmer seasons provide an ideal environment for mosquito breeding and population expansion, as the favorable conditions during these periods foster their increased prevalence. As noted by [60], mosquitoes exhibit increased feeding frequency and faster digestion in warmer climates, increasing the probability of laying more eggs. Additionally, agricultural activities during the summer and autumn seasons, which include irrigation may also provide breeding habitats for females to lay their eggs and for laval development as well as sources of nectar [61]. Conversely, colder winter temperatures decrease mosquito activities due to their cold-blooded nature [21], and their oviposition activity decreases during winter as *An. arabiensis* does not oviposit in dry and cold conditions [62]. The dry conditions in winter further limit mosquito breeding habitats as there is no rainfall contributing to a significant reduction in mosquito populations [63].

Mosquito density also showed monthly variations, with distinct peaks and lows observed throughout the year. Although not as consistent, the months from October to March had the highest densities, particularly in November and March. The *An. arabiensis* population gradually declines in April–May and plummets from June to August, similar to previous studies [11,64,65,66,67]. The abundance peaks coincide with the wet rainy season, while low densities occur in the dry winter season and towards spring. This aligns with *Anopheles* mosquito breeding patterns which thrive in warm rainy conditions [21,68]. The low peaks in winter months may result from low temperatures which inhibit mosquito activities [69]. Monthly variation could also be due to changes in trap numbers, possibly from damage or theft, leading to a reduction in the total collections.

The analysis of *An. arabiensis* density variations by area of collection showed that Section 2 had the highest collections throughout all seasons compared to the other two sections, likely due to its extensive coverage of the area exposed to a wetland. Section 8 had the least mosquito collections, potentially due to its relatively small area coverage of the wetland and vegetation removal in 2015, leading to the drying up of the portion of the wetland in Section 8, as documented by [11]. Wetlands, particularly mashes and swamps have traditionally been known to harbor mosquitoes [70,71]. They provide essential aquatic environments for larvae growth stages and nectar sources for the adults [72], but the reduction in wetland vegetation can result in drying, subsequently shrinking the wetland and reducing breeding sites and nectar availability [70].

The consistent abundance of *An. arabiensis* mosquito suggests that the current vector control efforts have reached a plateau, necessitating a complementary strategy such as the Sterile Insect Technique. Based on the population density recorded over the years the SIT is more suitable because it works best under low population density [58]. To enhance the SIT efficiency, it is recommended that wild males are supposed to be less than the sterile males, with a recommendation of 10 sterile to one wild male mosquito. This could potentially result in the eradication of the target species within 12 generations [73].

### 4.2. Impact of Climatic Factors on Anopheles arabiensis Density

Strong associations between mosquito populations and climatic variables have been investigated previously [19,20,21,22,24,74]. This study investigated the impact of four climatic variables (rainfall, temperature, wind speed, and relative humidity) on mosquito populations’ abundance. The wavelength cross-coherence and the lagged effect between mosquito abundance and climatic factors at different time frames were also explored. The results indicate that *An. arabiensis* in the Mamfene area has a varying degree of association and influence with rainfall, temperature, wind speed, and relative humidity.

Rainfall has been cited as a major driver for temporal dynamics in mosquito population abundance, influencing the aquatic stage of the mosquito life cycle such as the laying of mosquito eggs, the development of larvae, and the maturation into adults [24,26,74], although the exact type of association is influenced by several interacting factors such as the topology, elevation, aspect, rainfall intensity, duration, cumulative rainfall effects, habitat type as well as other climatic variables at play [25,75]. In this study, rainfall showed a negative significant correlation with *An. arabiensis* density at unlagged time, but a positive correlation at a lag time of 1–2 months. This suggests that excessive rainfall may wash away breeding sites and larvae initially, but subsequently creates aquatic conditions for oviposition and larval development over time [24]. This lag corresponds to the optimal duration for mosquito development from egg to adult, which is typically 10 days or more for anophelines [26]. These results are consistent with the findings of [76] where larvae were only found two to three weeks after rains but larvae were not found in areas where it had recently rained. Similarly, [24] found correlations between *An. arabiensis* and rainfall with a 0–90 days time lag. Additionally, a study by [25], in Kenya found also a significant association between anopheline density and rainfall 1–2 month lag although a peak was observed at 11 days.

Temperature has been reported to be the key climatic factor driving *Anopheles* mosquito population dynamics [28]. It has an inhibitory effect on mosquito life cycles whereby it influences the survival and host-seeking behavior of adult mosquitoes [30,63,77]. We established positive correlations between *An. arabiensis* density and temperature whereby the results showed that an increase in temperature leads to an increase in *An. arabiensis* density with a lag of 0–3 months. The in-phase relationship also confirmed that temperatures lead to high *An. arabiensis* density. Our findings suggest that increased mean temperatures for up to 3 months are correlated with the increased occurrence of *An. arabiensis* since mosquitoes are sensitive to temperature throughout their life cycle [28]; therefore, it has a longer lag period of influence. These findings are in line with [21], who articulate that higher temperatures favor higher transition rates between the mosquito stages, therefore, encouraging higher *An. arabiensis* density. Additionally, temperature is related to other climatic variables like humidity and rainfall being highly influential on mosquito dynamics. Higher correlations and associations might be due to the favorable temperature conditions offered by the study area throughout the year (mean of 38.4 °C) [21]. However, the findings of this study are contradictory with some existing studies [24,78] in which they found that increased temperatures have a negative impact and weaker association with the survival of mosquitoes. This might be because high temperatures may affect the water quality for the larval habitats where mosquitoes breed as well as causing high evaporation thereby reducing the size and occurrence of larval habitats [25].

The study showed that wind speed has a negative impact on *An. arabiensis* abundance. The time-lagged findings indicated a negative correlation at lag 0. These findings suggest that a high wind speed causes a decrease in *An. arabiensis* populations, similar to other findings [41,79,80]. This might be because the wind speed may cause water waves that can wash away larvae and pupae from their breeding habitat. In contrast, results from [81], reveal that wind speed has a direct significant correlation with mosquitoes and with Culex mosquitoes in particular [79]. This might be due to its influence on the direction and speed in which mosquitoes fly to the odor sources [43].

Relative humidity also plays a significant role in mosquitoes’ survival. The findings of this study showed that humidity has an in-phase relationship with *An. arabiensis* density. An increase in humidity increases mosquito abundance. This might be because mosquitoes become more active when humidity rises [82]. The results showed positive correlations with humidity at lag 0 and 3 months lags. This might be because the humidity is related to the survival as well as the hatching of mosquitoes’ eggs hence its significance throughout the mosquito life-span [77]. In support of these findings, [20] confirmed that elevated humidity fosters favorable conditions for increased mosquito abundance. Specifically, they noted that humidity levels reaching 85% offered the most conducive environment for mosquito collections, thereby signifying its importance in influencing mosquito trends. It is essential to consider these weather conditions, particularly for sterile male releases to ensure that they survive in the environment. The forecasted results showed that *An. arabiensis* density will continue to be prevalent throughout the months over the next 3 years that were predicted.

## 5. Conclusions

The research provided valuable baseline information about the *An. arabiensis* population dynamics, which is crucial for planning an effective SIT program. The study indicates that *An. arabiensis* remains the primary malaria vector in the study area, with its population fluctuating seasonally, peaking during the wet season and declining during the dry season. Climate variables were found to have varying impacts on the distribution and abundance of *An. arabiensis.* The models used offered a powerful tool to better understand the precise effect of each climatic factor in determining the distribution and influencing the temporal population dynamics of *An. arabiensis* species Mamfene, South Africa.

Although the study provides valuable insights into potential associations between mosquito density and climatic factors, other variables like ecological characteristics, vegetation conditions, and land cover, could also influence *An. arabiensis* abundance which needs to be considered in future studies. Further research and ongoing surveillance may be needed to monitor and adapt control measures to the everchanging mosquito populations and environmental conditions. It is imperative to continue developing comprehensive models that incorporate all relevant variables, coupled with spatial analysis to identify mosquito hotspots and coldspots and consequently tailor malaria vector control programs.

## Figures and Tables

**Table 1:** *Anopheles* mosquitoes collected between February 2014 and December 2019 from the three sentinel sections in Mamfene, northern KwaZulu-Natal, South Africa, using clay pots, stratified by species and collection method. NB: Percentages are calculated based on totals for each row.

Species Collected		Number Collected Using Clay Pot *n* (%)	Number Collected Using Other Collection Methods *n* (%)	Total Collected (*n*)
*An. gambiae* complex	*An. arabiensis*	3826 (29%)	9234 (71%)	13,060
	*An. gambiae*	1 (50%)	1 (50%)	2
	*An. merus*	362 (79%)	99 (21%)	461
	*An. quadriannulatus*	56 (72%)	22 (28%)	78
	^1^ No ID and Blanks	1494 (60%)	1006 (40%)	2500
Sub-total		5739	10,362	16,101
*An. funestus* group	*An. leesoni*	87 (72%)	34 (28%)	121
	*An. parensis*	647 (65%)	345 (35%)	992
	*An. rivulorum*	202 (76%)	63 (24%)	265
	*An. vaneedeni*	235 (79%)	62 (21%)	297
	^2^ No ID and blanks	970 (70%)	412 (30%)	1382
Sub-total		2141	916	3057
Other anopheline species	*An. rufipes*	111 (69%)	49 (31%)	160
	*An. coustani*	50 (63%)	29 (37%)	79
	*An. demeilloni*	7 (70%)	3 (30%)	10
	*An. maculipalpis*	5 (50%)	5 (50%)	10
	*An. marshallii group*	308 (74%)	107 (26%)	415
	*An. pharoensis*	12 (63%)	7 (37%)	19
	*An. pretoriensis*	43 (70%)	18 (30%)	61
	*An. squamosus*	2 (17%)	10 (83%)	12
	^3^ No ID, blanks and others	587 (74%)	203 (26%)	790
Sub-total		1125	431	1556
Total collections from all collection methods for all species				20,714

^1^ Mosquitoes morphologically identified as belonging to the *An. gambiae* complex but failed to be determined to species level using PCR. ^2^ Mosquitoes morphologically identified as belonging to the *An. funestus* group but failed to be determined to species level using PCR. ^3^ Mosquitoes morphologically identified as anophelines but failed to be determined to species level using morphological keys which might be due to damaged morphological key features.

**Table 2:** Summary of *Anopheles arabiensis* collected using clay pots from Mamfene, KwaZulu-Natal between January 2014 and December 2019 stratified by year, section, and season. NB: the number of *Anopheles arabiensis* shown for each of the seasons refers to the sum of the respective seasons for all the years in the study period (2014–2019). The percentages are based on the column totals.

Variable		Number of *An. arabiensis* Collected Per Section, N and Density Presented in (y)			Total Number of *An. arabiensis* Collected, N (Relative Abundance %)
		2	8	9	
Year	2014	138 (5.8)	44 (3.1)	53 (5.1)	235 (6.1%)
	2015	170 (2.3)	203 (3.0)	122 (2.7)	495 (12.9%)
	2016	482 (6.9)	145 (3.3.)	277 (3.5)	904 (23.6%)
	2017	213 (8.2)	39 (2.4)	99 (3.1)	351 (9.2%)
	2018	110 (1.9)	43 (1.5)	96 (2.2)	249 (6.5%)
	2019	373 (3.6)	199 (3.1)	1020 (6.2)	1592 (41.6%)
Sub-total		1486	673	1667	3826
Season	Summer	513 (7.3)	166 (4.4)	612 (5.5)	1291 (33.7%)
	Winter	354 (3.9)	220 (2.9)	320 (4.1)	894 (23.4%)
	Autumn	340 (11.9)	199 (5.6)	504 (8.3)	1043 (27.3%)
	Spring	279 (5.6)	88 (3.6)	231 (4.7)	598 (15.6%)
Sub-total		1486	673	1667	3826
Overall collections total		1486 (39%)	673 (18%)	1667 (44%)	3826

**Table 3:** Association between *Anopheles arabiensis* density and lagged climatic variables measured by Pearson’s correlation coefficient.

Lagged Period	Mean Temperature	Rainfall	Wind	Average Humidity
−3 month lag	0.123	−0.203	−0.349	0.261
−2 month lag	0.196	−0.097	−0.189	0.050
−1 month lag	0.433	*0.298*	0.055	−0.182
Unlagged (lag 0)	*0.537*	−0.155	−*0.466*	*0.495*
1-month lag	0.477	0.247	0.021	0.478
2-month lag	0.473	0.235	0.066	0.456
3-month lag	0.147	0.045	0.089	−0.371

Italic: maximum correlation.

## Data Availability

The mosquito data used in the analysis in this manuscript have been sourced from the Sterile Insect Technique database from the Vector Control Research Laboratory, NICD. The meteorological data was obtained from the South African Weather Service (SAWS).
